# The position of renal denervation in treatment of hypertension: an expert consensus statement

**DOI:** 10.1007/s12471-022-01717-4

**Published:** 2022-08-24

**Authors:** V. J. M. Zeijen, A. A. Kroon, B. H. van den Born, P. J. Blankestijn, S. C. A. Meijvis, A. Nap, E. Lipsic, A. Elvan, J. Versmissen, R. J. van Geuns, M. Voskuil, P. A. L. Tonino, W. Spiering, J. Deinum, J. Daemen

**Affiliations:** 1 Department of Cardiology, Erasmus University Medical Center, Rotterdam, The Netherlands; 2 Department of Internal Medicine, Maastricht University Medical Center & Cardiovascular Research Institute Maastricht, Maastricht, The Netherlands; 3 Department of Vascular Medicine, Amsterdam University Medical Center, Amsterdam, The Netherlands; 4 Department of Nephrology and Hypertension, University Medical Center Utrecht, Utrecht, The Netherlands; 5 Department of Cardiology, Amsterdam University Medical Center, Amsterdam, The Netherlands; 6 Department of Cardiology, University Medical Center Groningen, Groningen, The Netherlands; 7 Department of Cardiology, Isala Heart Center, Zwolle, The Netherlands; 8 Department of Internal Medicine, Erasmus University Medical Center, Rotterdam, The Netherlands; 9 Department of Cardiology, Radboud University Medical Center, Nijmegen, The Netherlands; 10 Department of Cardiology, University Medical Center Utrecht, Utrecht, The Netherlands; 11 Department of Cardiology, Catharina Hospital, Eindhoven, The Netherlands; 12 Department of Vascular Medicine, University Medical Center Utrecht, Utrecht, The Netherlands; 13 Department of Internal Medicine, Radboud University Medical Center, Nijmegen, The Netherlands

**Keywords:** Netherlands, Consensus, Sympathectomy, Hypertension, Patient care

## Abstract

Hypertension is an important risk factor for cardiovascular disease. In the Netherlands, there are approximately 2.8 million people with hypertension. Despite treatment recommendations including lifestyle changes and antihypertensive drugs, most patients do not meet guideline-recommended blood pressure (BP) targets. In order to improve BP control and lower the risk of subsequent cardiovascular events, renal sympathetic denervation (RDN) has been introduced and studied as a non-pharmacological approach. While early data on the efficacy of RDN showed conflicting results, improvements in treatment protocols and study design resulted in robust new evidence supporting the potential of the technology to improve patient care in hypertensive subjects. Recently, 5 randomised sham-controlled trials demonstrated the safety and efficacy of the technology. Modelling studies have further shown that RDN is cost-effective in the Dutch healthcare setting. Given the undisputable disease burden along with the shortcomings of current therapeutic options, we postulate a new, clearly framed indication for RDN as an adjunct in the treatment of hypertension. The present consensus statement summarises current guideline-recommended BP targets, proposed workup and treatment for hypertension, and position of RDN for those patients with primary hypertension who do not meet guideline-recommended BP targets (see central illustration).

## Clinical relevance

Hypertension is a major public health problem prevalent in 18% (2.8 million people) of all Dutch adults [1, 2]. Previous studies showed that guideline-recommended blood pressure (BP) targets were only achieved in 28.4 to 41.8% of all patients [3–5]. As a result, hypertension accounts for 6.7% of all disability-adjusted life years (DALYs) in the Netherlands [6]. More specifically, the population attributable risk (PAR) of hypertension was substantial for both stroke (38.8%) and myocardial infarction (20.5%) [7, 8].

Current guidelines recommend lifestyle modifications and antihypertensive drugs as first-line therapy of patients with hypertension [9, 10]. This standard therapy has proven to reduce cardiovascular risk by 10 to 20% for every 5 to 10 mm Hg decrease in systolic office BP (sOBP) respectively [11–13]. This effect was consistent among several age subgroups, including octogenarians, in whom intensive BP lowering using standard pharmacotherapy has been linked to an increased risk of renal adverse events [14]. Nevertheless, non-adherence to antihypertensive drugs proved to jeopardise BP control in 28 to 50% of all patients [15–17]. A significantly increased adverse cardiovascular event risk in non-adherent patients demonstrates the importance of proper therapy adherence [18–20]. Several methods to improve drug adherence have been proposed and include, but are not limited to, patient education, diaries, apps and alarms. More recently, therapeutic drug monitoring has been tested as a promising and reliable technology to define non-adherence [21]. However, as a BP-lowering effect of therapy adherence testing has not been demonstrated, the search for novel non-pharmacological treatment options to improve BP control is warranted.

Pre-clinical trials demonstrated that the sympathetic nervous system (SNS) plays an important role in the pathophysiology of hypertension [22, 23]. In fact, the BP-lowering effect of beta-blockers, alpha-blockers, and centrally acting drugs has been directly linked to decreased SNS tone [24]. Disruption of renal nerve activity specifically has been shown to prevent, delay or reduce the magnitude of hypertension in a wide variety of animal models [25, 26]. Renal sympathetic denervation (RDN) targets sympathetic nerves at the renal artery level and proved to reduce sympathetic overactivity and lower BP in the absence of side effects as sometimes observed in patients on antihypertensive (sympatholytic) pharmacotherapy [27].

Next to RDN, alternative device-based techniques such as baroreflex activation, baroreflex amplification and cardiac neuromodulation have shown promising results in proof-of-principle studies [28]. Future studies need to identify the potential role of these technologies in hypertension treatment. In parallel, new pharmaceutical agents for the (personalised) treatment of hypertension are being developed. The agents studied involve, but are not limited to, sodium-glucose cotransporter 2 (SGLT2) inhibitors, angiotensin receptor II blocker—neprilysin inhibitors (ARNI) and small-interfering RNAs, which have demonstrated promising results in preclinical and early-phase clinical studies [29]. However, as of to date, sham- or placebo-controlled randomised clinical trials (RCTs) supporting the efficacy and safety of these novel treatment options are lacking.

The previous consensus statement on RDN published in 2014 suggested RDN to be a promising therapy to lower BP. However, following the neutral result of the SYMPLICITY HTN‑3 trial, the document also made clear recommendations on the restricted use of RDN for those patients followed in context of dedicated clinical studies [30]. Following the publication of this pivotal consensus statement, a substantial amount of new evidence has emerged refining the safety and efficacy of RDN. In the present consensus document we provide an overview of the scientific advances in the management of hypertension, including guideline-recommended BP targets, proposed workup and treatment, and position of RDN for those patients with primary hypertension who do not meet guideline-recommended BP targets.

## Current treatment targets and proposed workup

According to current guidelines formulated by the European Societies of Cardiology (ESC) and Hypertension (ESH), hypertension is diagnosed in patients with sOBP ≥ 140 mm Hg and/or diastolic office BP (dOBP) ≥ 90 mm Hg [9]. Furthermore, ambulatory BP (ABP) monitoring is recommended to confirm hypertension according to the following criteria: systolic ABP (sABP) ≥ 130, 135 or 120 mm Hg and/or diastolic ABP (dABP) ≥ 80, 85 or 70 mm Hg for mean 24-hour, daytime and nighttime ABP respectively [9]. Workup includes cardiovascular risk profiling and evaluation of the presence of hypertension-mediated organ damage (HMOD) and clinical clues for secondary causes of hypertension [9]. HMOD is defined as structural and functional changes in arteries and/or organs exposed to (long-standing) hypertension, such as the heart, brain, retina and kidneys, and is strongly related to future adverse cardiovascular events [9]. The most common secondary causes are considered obstructive sleep apnoea syndrome (OSAS) and renal parenchymal, renovascular or endocrine diseases [9]. Initiation of stepwise antihypertensive drug treatment is recommended irrespective of cardiovascular risk in all patients who do not meet BP targets despite lifestyle recommendations and should target the most important pathways that lead to hypertension [9]. Routine advised drug therapy therefore consists of (a combination of) calcium channel blockers (CCB), renin-angiotensin inhibiting agents and diuretics [9]. In patients not meeting their BP targets, the addition of spironolactone or other diuretic, alpha-blocker or beta-blocker is recommended on top of the triple-pill combination mentioned above [9].

Whereas the Dutch guidelines on hypertension treatment are largely in agreement with European guidelines, the Dutch guidelines have a more pronounced focus on guiding treatment in light of the patient’s individual overall cardiovascular risk profile and thereby directly impact the recommended urgency of initiation and intensification of antihypertensive treatment [9, 10].

## Currently available evidence on RDN

### Treatment efficacy

RDN is at present one of the most widely studied invasive approaches for the treatment of hypertension. With the first generation of RDN catheters, varying effect magnitudes of BP reduction in patients who underwent RDN were reported [31–33]. Non-standardised medical treatment, changes in antihypertensive medication throughout the course of the trials and suboptimal denervation procedures were soon recognised as major factors complicating device-based antihypertensive therapy research [34]. Addressing these limitations in improved study protocols and new denervation techniques, five proof-of-principle RCTs proved the overall efficacy and safety of RDN in patients on and off antihypertensive medication [35–40].

Two RCTs were performed in patients taken off antihypertensive drugs in a well-controlled setting to determine treatment efficacy of RDN in the absence of any antihypertensive drug effects [35, 37, 39]. These studies enrolled patients with uncontrolled, mild to moderate hypertension and a low cardiovascular risk [35, 37, 39]. The SPYRAL HTN-OFF MED trial (*n* = 331) investigated the effect of radiofrequency (RF) RDN (using the Medtronic Symplicity Spyral multi-electrode catheter (Medtronic, Galway, Ireland)) whereas the RADIANCE HTN SOLO trial (*n* = 146) evaluated the effect of ultrasound (US) RDN (using the Paradise Renal Denervation System (ReCor Medical, Palo Alto, CA, USA)) [35, 37, 39]. Both RDN techniques proved efficacious in achieving a significant drop in sABP (−3.9 to −6.3 mm Hg), dABP (−2.6 to −4.4 mm Hg), sOBP (−6.5 to −7.7 mm Hg) and dOBP (−4.1 to −4.9 mm Hg) at two to three months compared with a sham-control arm [35, 39]. In the RADIANCE HTN SOLO trial, standardised antihypertensive drug treatment was introduced after assessment of the primary endpoint at two months. At six months, the efficacy of RDN was confirmed as patients who underwent RDN had lower sABP (−4.3 mm Hg) and sOBP (−3.7 mm Hg) on a lower burden of antihypertensive drugs compared with control patients [41].

Next, RDN was evaluated with a similar level of scrutiny in patients on antihypertensive drugs in three RCTs [36, 38, 40]. The DENERHTN trial (*n* = 106) evaluated the effect of RDN in patients not meeting BP targets despite the use a standardised, triple-pill antihypertensive drug regimen (indapamide, ramipril (or irbesartan) and amlodipine) [36]. Patients were randomised to RDN (using the Medtronic Symplicity Flex uni-electrode RF catheter) plus standardised stepped antihypertensive treatment (SSAHT) or SSAHT alone [36]. The SSAHT uptitration scheme consisted of spironolactone, bisoprolol, prazosin and rilmenidine, accordingly [36]. RDN on top of SSAHT resulted in an additional reduction in sABP (−5.9 mm Hg) compared with SSAHT alone at six months [36]. These findings were confirmed by the preliminary results of the SPYRAL HTN-ON MED (*n* = 80) trial that investigated the effect of RF-RDN (using the Medtronic Symplicity Spyral multi-electrode catheter) in patients not meeting BP targets while on a non-standardised, stable regimen of one to three antihypertensive drugs [38]. RDN proved to effectively lower sABP (−7.0 mm Hg), dABP (−4.3 mm Hg), sOBP (−6.6 mm Hg) and dOBP (−4.2 mm Hg) at six months as compared with sham-control [38]. These results were confirmed in the RADIANCE TRIO trial which evaluated the efficacy of US RDN (using the Paradise Renal Denervation System) in patients on a standardised triple-pill antihypertensive drug regimen (amlodipine, valsartan and hydrochlorothiazide) [40]. The study demonstrated a significant reduction in sABP (−4.5 mm Hg) and sOBP (−7.0 mm Hg) two months after RDN compared with sham-control, whereas dABP and dOBP did not differ between both groups [40]. In contrast, no significant reduction in sABP post RDN compared with sham-control was observed in the REQUIRE trial which refrained from standardising antihypertensive therapy and adherence testing [42]. When comparing US-RDN to RF-RDN treatment, the RADIOSOUND study demonstrated that US-RDN results in similar BP reductions as RF-RDN including any accessory renal arteries [43].

Whereas significant mean BP reductions have been observed post RDN, the treatment effect in individual patients showed substantial heterogeneity, with approximately one out of three patients exhibiting no significant BP response to RDN [35, 38, 44, 45]. Unfortunately, as of to date, consistent predictors of treatment response have not yet been identified [28].

Whereas more pragmatic trials with more lenient entry criteria are needed, the global SYMPLICITY registry reported significant and sustained BP reductions in real-world patients [46].

### Treatment durability

Demonstration of durability of the BP response following interventional procedures is challenged by changes in medications, coexisting illness and patient behaviour (e.g. weight loss, exercise and diet). Animal studies suggest the potential of renal nerve regeneration over time [47, 48]. However, renal hormone excretion was only partially restored [47]. Whereas evidence on this phenomenon lacks in humans, registry studies confirmed a durable sABP-lowering effect of −8.0 and −20.9 mm Hg up until three and five years after RDN respectively [46, 49]. In the RADIANCE HTN SOLO study, sOBP was lower in the RDN group (−5.9 vs. −4.3 mm Hg) while on less medication compared with the control group 12 months post randomisation [50]. The 3‑year results of the SPYRAL HTN-ON MED study demonstrated a persistent reduction in sABP of −10.0 mm Hg post RDN as compared with sham-control, which could not be explained by differences in prescribed antihypertensive drug regimen or therapy adherence [51].

### Treatment safety

Several clinical trials confirmed an excellent safety profile of RDN with no major procedure-related adverse events or relevant decrease in renal function [35, 36, 38–40, 46, 52]. Significant renal artery stenosis is observed in only 0.5% of all patients with 80% of all cases discovered within one year after RDN [53].

### Clinical outcome data

At present, no direct evidence is available on the effectiveness of RDN in lowering the risk of cardiovascular events. Nevertheless, the BP reduction achieved by RDN will likely result in comparable cardiovascular risk reduction as achieved by conventional antihypertensive drug treatment [12, 13]. Indirect evidence on a potential additive effect of RDN on top of medical therapy on clinical outcome can be derived from the positive effect of RDN on regression of HMOD and ABP patterns in hypertension following RDN [54]. Whether the documented BP-lowering effects are persistent through long-term follow-up and lead to improved cardiovascular endpoints must be investigated in future clinical studies [55].

### Cost-effectiveness

Several studies on health decision modelling have reported modelled incremental cost-effectiveness ratios (ICER) for RDN ranging from € 1,474 to € 6,573 per quality-adjusted life year (QALY) [56–58]. The only Dutch study investigating the cost-effectiveness of RDN demonstrated the lowest ICER from all studies (€ 1,474 per QALY) [58]. The latter shows RDN is considered a cost-effective treatment for all common willingness-to-pay thresholds in the Netherlands [59]. As all published evidence on cost-effectiveness on RDN is currently based on first generation trials, the cost-effectiveness analyses of second generation trials are eagerly awaited.

### Patient preference

In patients who require antihypertensive treatment, there is a profound interest for non-pharmacological, invasive treatment options over taking drugs on a daily base. About 8.2% of all patients in the United States would be willing to trade two years off their lives to avoid taking any drugs for cardiovascular prevention [60]. For RDN specifically, 28% of all drug-treated uncontrolled hypertensive German patients would prefer RDN over further intensification of drug therapy [61]. In a recent multi-country (including the Netherlands) 15-day social media campaign recruiting hypertensive patients for a novel RDN trial, 12,000 individuals clicked on the advertisement which resulted in over 400 registrations for that particular trial [62].

### Ongoing studies

At present, several new studies investigating the safety and efficacy of different RDN technologies are ongoing. For radiofrequency RDN, the SPYRAL ON-MED study will focus on the effect of RDN on top of antihypertensive therapy (ClinicalTrials.gov Identifier: NCT02439775). With respect to ultrasound RDN, the RADIANCE II pivotal study will focus on the treatment effect in absence of antihypertensive medication (ClinicalTrials.gov Identifier: NCT03614260). In parallel, the TARGET BP I (ClinicalTrials.gov Identifier: NCT02910414) and TARGET BP OFF-MED (ClinicalTrials.gov Identifier: NCT03503773) trials currently investigate RDN using perivascular alcohol infusion in patients on and off antihypertensive medication respectively.

### Topics to be addressed in future research

In the upcoming years, a shift from proof-of-concept trials to pragmatic real-world RDN studies might be expected. Registries including large numbers of patients will reveal more information about long-term efficacy and safety of the technology. To allow for poolability of long-term data from different studies, trials will have to be designed in a standardised fashion with respect to inclusion criteria and outcome measures. As such, the upcoming Hypertension Academic Research Consortium (HARC) statement will provide further guidance on the matter [63]. In parallel, more insights into predictors of RDN success are required to facilitate adequate selection of patients who are most likely to benefit from treatment. Previous studies have identified nighttime sABP as well as its variability, 24-hour dABP, 24-hour heart rate, pulse wave velocity, central pulse pressure, gender and a history of diabetes mellitus as predictors of the treatment effect of RDN, but caution has to be applied as these predictors were mostly detected in retrospective post-hoc analyses [64–69]. Finally, there is a growing interest in measuring and defining technical and procedural success. As such, renal artery nerve mapping has shown to be a safe and feasible technique [70, 71]. This procedure allows for measuring the effect of renal nerve stimulation on BP pre-RDN, which was shown to be correlated to decrease in ABP afterwards [72].

### Evidence for treatment indications outside of hypertension

This current consensus statement focusses on RDN as a well-investigated, promising therapy for patients with hypertension. In parallel, the safety and efficacy of RDN for alternative indications and conditions associated with sympathetic overactivity, such as kidney failure, kidney-related pain syndromes, atrial fibrillation, ventricular arrhythmias, heart failure, insulin resistance, metabolic syndrome and OSAS, have been studied [63]. The consortium believes more data is needed to decide on the role of RDN in the treatment of the diseases mentioned above and advises to refrain from RDN treatment in patients with these conditions outside well-controlled study settings.

## Dutch perspective for RDN

### Society statements

**Fig. 1:**
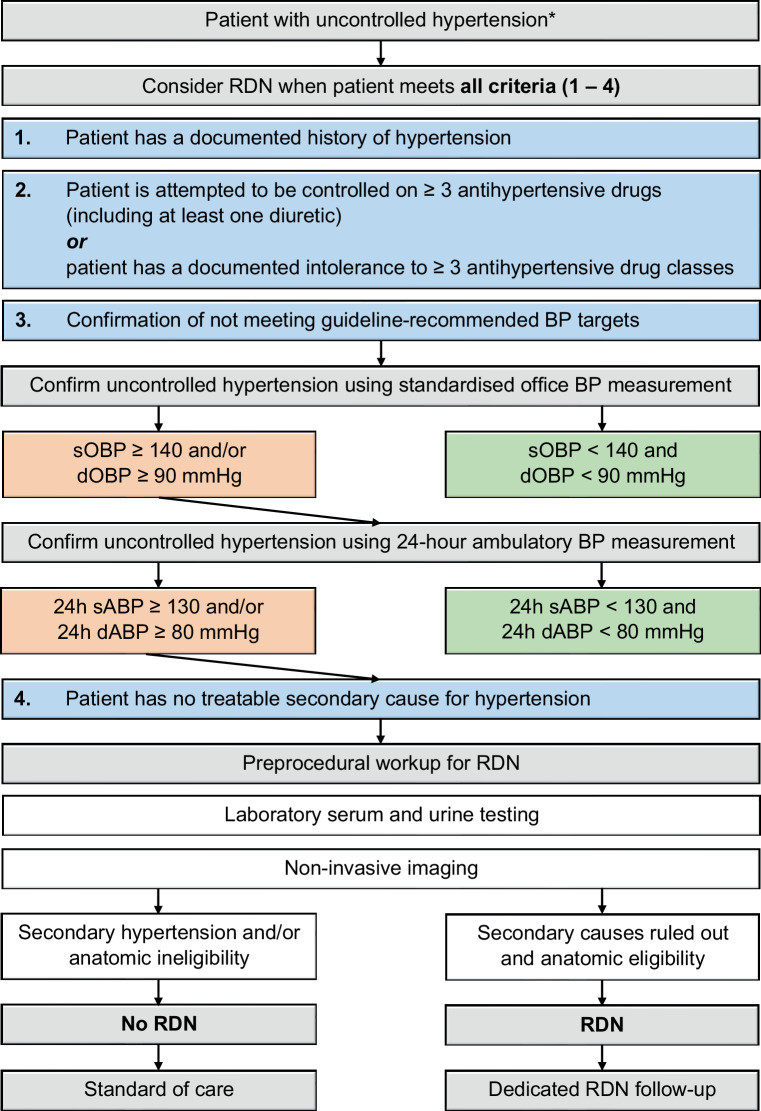
Flowchart for the positioning of RDN in clinical practice. (*According to 2018 ESC/ESH Guidelines for the management of arterial hypertension [9]. *BP* blood pressure, *sOBP* systolic office blood pressure, *dOBP* diastolic office blood pressure, *sABP* systolic ambulatory blood pressure, *dABP* diastolic ambulatory blood pressure, *RDN* renal sympathetic denervation, *ESC* European Society of Cardiology, *ESH* European Society of Hypertension)

The latest statement on the position of RDN in the treatment of hypertension was published in 2014 [30]. Since then, a substantial body of evidence has emerged positioning the role of RDN in patients on and off antihypertensive medication. Consequently, we have comprised a new consortium, consisting of Dutch experts in the field of hypertension with a background in vascular medicine, nephrology and cardiology. Throughout multiple meetings and several rounds of feedback, this consortium reviewed the evidence available and discussed the position of RDN in the Netherlands, including treatment indications, patient work-up and follow-up. Based on the outcome of these meetings, the consortium considers RDN to be an adjunctive treatment modality with proven efficacy that can help improve BP control in patients with uncontrolled hypertension despite routine guideline-recommended medical therapy and for patients who are intolerant to three or more classes of antihypertensive drugs (Fig. 1). Before RDN is considered, secondary causes of hypertension should be excluded and specific attention should be paid to therapy adherence, acknowledging the exponential increase in non-adherence in patients prescribed ≥ 4 drugs [73].

The consortium proposes the following treatment indication for the use of RDN in the treatment of hypertension in the Netherlands (all criteria need to be fulfilled):

1. Patient has a documented history of hypertension (according to current guidelines).
2. Patient was attempted to be controlled on three or more antihypertensive drugs (including at least one diuretic) for at least three months or has a documented intolerance to at least three different classes of antihypertensive drugs.
3. Patient does not meet guideline-recommended OBP targets (sOBP ≥ 140 mm Hg and/or dOBP ≥ 90 mm Hg) as confirmed by ABP measurement (24-hour sABP ≥ 130 mm Hg and/or dABP ≥ 80 mm Hg).
4. Patient has no treatable secondary cause for hypertension.

### RDN in clinical daily practice

When a patient is considered eligible for RDN, the consortium agrees on the need for extensive and standardised preprocedural screening [74]. Screening diagnostics will in any case consist of, but are not limited to, standardised OBP and ABP measurement, serum and urine laboratory testing and non-invasive imaging. ABP measurements have to be performed on top of standardised OBP measurements as ABP measurements are more closely correlated to cardiovascular risk than OBP measurements and should be used to rule out a white-coat hypertension [75, 76]. Serum (sodium, potassium, creatinine and renal function, haemoglobin, fasting glucose, HbA1c, fasting lipids, thyroid-stimulating hormone, renin and aldosterone) and urine (sodium, potassium, creatinine, protein and (micro-)albumin) laboratory testing will have to be performed to assess renal function, to evaluate existing HMOD, if any, and to detect potential secondary causes for hypertension. Especially, primary hyperaldosteronism should be ruled out using appropriate screening tests under standardised conditions consisting of measurement of plasma renin activity (or concentration) and serum aldosterone to calculate the aldosterone-to-renin ratio. Likewise, electrocardiography and echocardiography are recommended for the assessment of any HMOD. Renal imaging using either computed tomography angiography (CTA) or magnetic resonance angiography (MRA) should be performed to rule out renal artery stenosis, fibromuscular dysplasia or adrenal tumours, to confirm anatomic eligibility for RDN treatment (according to specific criteria per RDN device) and to facilitate procedural planning. Patients with renal artery abnormalities, history of nephrectomy, presence of a mono-kidney and pregnancy should not undergo RDN. Furthermore, little data is available on the safety and efficacy of RDN in patients with an estimated glomerular filtration rate (eGFR) < 30 ml/min/1.73 m^2^. As such, the use of RDN in these patients should be restricted to highly selected patients with therapy-resistant hypertension in whom there is multidisciplinary consensus on a lack of alternative options.

When the patient passed their preprocedural screening and anatomic eligibility is confirmed by conventional angiography, RDN has to be performed by certified operators in a catheterisation laboratory with the assistance of well-trained staff members according to local care. The consortium advises one night of hospital admission for all patients who underwent RDN. When no complications arise, patients will be discharged from the hospital the next morning. Prescription of aspirin up until one month post RDN should be considered.

Following treatment, the consortium recommends routine follow-up up to five years. The advised scheme consists of scheduled outpatient clinic visits at 1‑3-6 months and 1‑2-3-4-5 years after RDN. During all visits standardised OBP measurement should be performed, as well as ABP measurement at all visits from the third month’s visit onwards. In addition, serum and urine laboratory tests including renal function assessment are recommended to be performed during all visits. Finally, there should be a low threshold for repeat renal artery imaging using CTA or MRA at any stage during follow-up in case of persistent hypertension or a clinically relevant decline in renal function.

Follow-up (up to 5 years) at a dedicated hypertension clinic is advised for adequate registration of major cardiovascular events. Initiatives for coordinated national data registration on the use of RDN are currently being explored.

### Reimbursement

From 2013, RDN was subject to conditional reimbursement in the Netherlands. However, in December 2016 the Dutch National Health Care Institute (ZIN) decided not to continue reimbursement for RDN for the treatment of (therapy-resistant) hypertension following publication of the negative results of the SYMPLICITY HTN‑3 and Sympathy trials [33, 77]. Since then, the use of RDN in the Netherlands has been restricted to clinical trial settings. Following the publication of several more recent sham-controlled RCTs, demonstrating both safety and efficacy of RDN, renewed discussions have been initiated in an attempt to get reimbursement for RDN. The latter is a joined effort involving the Dutch Societies of Cardiology (NVVC), Internal Medicine (NIV) and Radiology (NVvR) together with other stakeholders and industry partners.

## Conclusions

Since the publication of the previous Dutch consensus statement on the implementation of RDN, dedicated evidence confirming efficacy, safety and cost-effectiveness of this procedure has been published. Blood pressure reductions observed across these studies proved to be consistently greater than 5 mmHg sOBP, to which a clinically meaningful reduction in cardiovascular events can be expected. Based on extensive review of the recent clinical evidence, including five RCTs, we conclude established treatment indications are available for which RDN could improve routine clinical practice. We believe that RDN could be a valid adjunct treatment option in patients with primary hypertension who do not meet guideline-advised OBP and ABP criteria despite the use of three or more antihypertensive drugs (including a diuretic), or in those with a documented intolerance to at least three different antihypertensive drug classes. Careful preprocedural workup including multimodal diagnostic testing as well as postprocedural follow-up visits are strongly recommended.
